# Changes in seroprevalence of hepatitis B surface antigen and epidemiologic characteristics in the Republic of Korea, 1998-2013

**DOI:** 10.4178/epih/e2015055

**Published:** 2015-12-23

**Authors:** Hyerin Lee, Hyungmin Lee, Yumi Cho, Kyungwon Oh, Moran Ki

**Affiliations:** 1 Division of Health and Nutrition Survey, Centers for Disease Control and Prevention, Cheongju, Korea; 2 Department of Cancer Control and Policy, Graduate School of Cancer Science and Policy, National Cancer Center, Goyang, Korea

**Keywords:** Hepatitis B surface antigen, Seropositivity, Republic of Korea, Korea National Health and Nutrition Examination Survey, Epidemiologic characteristics

## Abstract

**OBJECTIVES:**

This study investigated changes in hepatitis B seroprevalence from 1998 to 2013, and to identify differences in epidemiologic characteristics between hepatitis B surface antigen (HBsAg)-positive and HBsAg-negative people.

**METHODS:**

HBsAg seropositive rates were compared by year, sex, and age using the blood test data from the periods I to VI (1998-2013) of the Korea National Health and Nutrition Examination Survey. Interviews and self-administered surveys were conducted to collect data on health behavior, quality of life, comorbidities, and health care utilization.

**RESULTS:**

HBsAg seropositive rates in the Republic of Korea decreased from 4.6% in 1998 to 2.9% in 2008, and then remained the same for the next five years. While seropositive rates by age were the highest at 35 to 39 years of age in 1998, it peaked at 50 to 54 years of age in 2013. HBsAg-positive people showed high values from two liver function tests, including glutamic-oxaloacetic transaminase and glutamic-pyruvic transaminase, and the prevalence rates of liver cirrhosis and liver cancer were also significantly high. Indices for health behavior and quality of life showed no significant differences between HBsAg-positive and HBsAg-negative people.

**CONCLUSIONS:**

While HBsAg seropositive rates tended to decline after 1998, there have been no significant changes over the most recent five years. We should focus on treatment of existing hepatitis B patients along with immunization programmes to prevent new hepatitis B infections. In addition, it may be necessary to encourage HBsAg-positive people to follow healthier life-styles in order to prevent further progression of hepatitis B to liver cirrhosis and liver cancer.

## INTRODUCTION

Infection by hepatitis B virus (HBV) is a major risk factor for chronic liver diseases and is also a serious public health problem because of its high incidence worldwide. According to a report by the World Health Organization, around two billion people in the world have been infected by HBV, and it was estimated in 2015 that approximately 250 million people die of diseases related to HBV infection every year [1,2]. Hepatitis B surface antigen (HBsAg) has been widely used for diagnosis of these HBV-related liver diseases. HBsAg positivity can be used to diagnose acute HBV infection and chronic hepatitis B for longer than six months [3]. Hepatitis B vaccination was introduced into the Republic of Korea (hereafter Korea) beginning in 1983, and a national mandatory immunization program for infants has been conducted since 1995. In addition, in order to prevent vertical infection, costs for a prevention program including hepatitis B vaccination and immunoglobulin of infants born to hepatitis B-infected mothers have been supported since July 2002 [4]. As a result of these various efforts, HBsAg seropositivity in the Korea are reported to decrease continuously [5]. However, since there has been no report on HBsAg seroprevalence in the Korea after 2005, the trend of HBsAg seropositivity needs to be investigated for the most recent 15 years.

Hepatitis B patient can maintain their health and break the chain of infection through regular liver function tests, timely treatments, and vaccinations of their families. Therefore, hepatitis B patient need to maintain a healthy lifestyle based on accurate knowledge; nevertheless, a recent study reported a low rate of proper health behaviors, such as no alcohol drinking, regular physical activity, and regular examinations [6]. Most chronic hepatitis patients had psychosocial stresses such as withdrawal from interpersonal relationships and depression [7-9]. Therefore, identifying these factors and to prepare an appropriate health management strategy is important.

Accordingly, this study aimed to investigate the national trend of HBsAg seroprevalence from 1998 to 2013 through the Korea National Health and Nutrition Examination Survey (KNHANES) and to compare clinical characteristics, health behavior, quality of life, comorbidities, and health care utilization depending on HBsAg positivity.

## MATERIALS AND METHODS

### Study population

KNHANES is an ongoing surveillance system that assesses the health and nutritional status of Koreans, monitors trends in health risk factors and the prevalence of major chronic diseases and provides data for the development and evaluation of health policies and programs in the Korea. KNHANES was first established in 1998 and conducted by Centers for Disease Control and Prevention. It was performed every three years for periods I to III (1998, 2001, and 2005); thereafter, it has been changed to a yearly survey system from 2007. Research subjects were selected by using two-stage stratified cluster sampling with the population and housing census data. Surveys were conducted with about 35,000 people for the periods I to III and about 10,000 people for the period IV to VI. However, the survey in 2007 was conducted with about 5,000 people for six months (July to December) [10].

HBsAg seropositivity were investigated using data from people 10 years or older who completed clinical examinations, but some data were surveyed only among people aged 19 or above. The final numbers of subjects for the HBsAg test were 9,771 people in 1998, 7,918 in 2001, 6,404 in 2005, 3,480 in 2007, 7,670 in 2008, 8,304 in 2009, 6,815 in 2010, 6,702 in 2011, 6,301 in 2012, and 5,938 in 2013.

### Health examination and interview

For analysis of HBsAg, about 15 mL of blood was collected and followed by separation of the serum within 30 minutes, which was then transferred to institution. Collected specimens were analyzed by electrochemiluminescence immunoassay within 24 hours. Analysis results were presented as presence or absence of HBsAg from 1998 to 2001 and as values from 2005 to 2013 [11], and variables for HBsAg positivity provided in the raw data were used for analysis of results. While the analyzers and reagents were different depending on testing year (Table 1), quality was maintained appropriately through internal and external quality management every year [12].

Among the health interview survey items, medical conditions (tuberculosis, hepatitis C, liver cirrhosis, and liver cancer), healthcare utilization, and quality of life were investigated via face-to-face interviews, whereas health behavior items such as smoking, alcohol use, physical activity, and mental health were investigated via a self-administration. “Current cigarette smoking” was defined as smoking at least five packs of cigarettes (100 cigarettes) in the life-time and currently smoking, and “high-risk drinking” was defined as having seven drinks or more (five drinks or more for female) of alcohol on the same occasion on each of two or more days a week. “Vigorous physical activity” was defined as participating in vigorous physical activity that take hard physical effort or make breathe much harder than normal at least 10 minutes at a time, for 20 minutes and more per day, three or more days per week during the last seven days. “Moderate to vigorous physical activity” was defined as participating in vigorous physical activity or moderate physical activity that take moderate physical effort or make breathe somewhat harder than normal at least 10 minutes at a time, for 30 minutes or more per day, 5 or more days per week during the last seven days. “Perceived stress” was defined as feeling stress ‘very much’ or ‘much’. “Feeling sad or hopeless” meant feeling sad or hopeless almost everyday for two or more weeks continuously that they stopped doing some usual activities during the last year.

“Health related quality of life” was measured by using EQ-5D (mobility, self-care, usual activities, pain/discomfort, and anxiety/depression) and EQ-VAS (vertical visual analogue scale), and EQ-5D index score was calculated using Korean population-based preference weights [13].

Hypertension was defined as when systolic blood pressure was 140 mmHg or higher, diastolic blood pressure was 90 mmHg or higher, or hypertension medicine was taken, and diabetes was defined when individuals had a 126 mg/dL or higher fasting blood glucose level, a diagnosis by physicians, were taking hypoglycemic agents, or using insulin injections. Prevalence of tuberculosis, hepatitis C, liver cirrhosis, and liver cancer were defined as respondent report of physician-diagnosed these conditions. Hospital utilization as an outpatient during the last two weeks, hospitalization for the last year, and cancer screening for the last two years were investigated. Unmet needs indicated adults who did not go to a hospital/clinic (excluding dental clinics) when a person needed medical care during the last year.

Statistical analysis was performed with SAS survey procedures version 9.4 (SAS Institute Inc., Cary, NC, USA) reflecting sample design and weight. HBsAg seropositive rates by year were compared after age-standardization based on the projected population in 2005. Unweighted total number and weighted % were calculated about clinical examination, health behavior, mental health, quality of life, comorbidity and health care utilization by HBsAg positivity. P-values were obtained by chi-square test for comparison of the proportions.

## RESULTS

### Hepatitis B surface antigen seropositivity by year and sex

**Figure 1.**
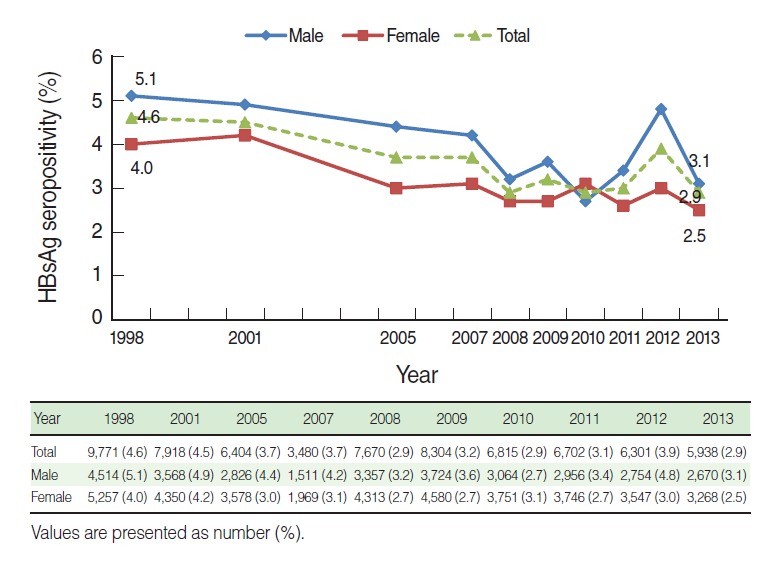
Hepatitis B surface antigen (HBsAg) seropositivity by sex and year among ≥10 years based on the Korea National Health and Nutrition Examination Survey I to VI (1998-2013), Republic of Korea. Age standardization method is applied using the 2005 population estimates.

The HBsAg seropositive rate of subjects 10 years or older was 4.6% (5.1% for male and 4.0% for female) in 1998, and tended to decrease as shown to 2.9% in 2008. Thereafter it remained at the same level, ending around 2.9% (3.1% for male and 2.5% for female) in 2013. The seropositive rate of males were at its lowest (2.7%) in 2010 and suddenly increased to 4.8% in 2012, while the trend in the remaining years was consistent with the general pattern. In contrast, the seropositive rate of females rapidly declined to 3.1% in 2005, and then remained between 2.7% and 3.1% without significant changes. Males consistently had a higher infection rate than females, with a gap of 1.1% in 1998, whereas it reduced to 0.6% in 2013 (Figure 1).

**Figure 2.**
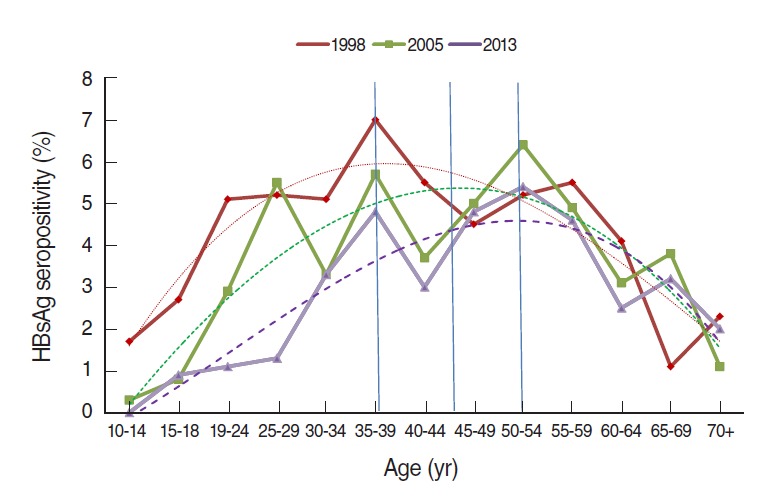
Hepatitis B surface antigen (HBsAg) seropositivity by age between 1998 and 2013, Republic of Korea. Dotted lines are order three polynomial trend line: R^2^=0.784 for 1998, 0.742 for 2005, and 0.812 in 2013.

HBsAg seropositivity results from 1998 to 2013 were compared by age. In order to test trends with minimizing variations that are caused by small numbers of subjects in each age group, order three polynomial trend lines with the highest goodness of fit were added. R2 values of trend lines were 0.784 in 1998 and 0.812 in 2013. Overall, seropositive rate increased and then decreased with age; however, the peaks appeared at age late 30s (35 to 39 years) in 1998 and age early 50s (50 to 54 years) in 2013. These results reflect the 15 year-interval between the two investigations. Seropositive rates were decreased by about 1% to 2% in all age cohorts over the 15 years. While seropositive rates in individuals in their late 50s or older were higher in 2013, those in their 10s and 20s were less than 1% and just over 1%, respectively (Figure 2).

### Clinical test results by hepatitis B surface antigen seropositivity

HBsAg-positive people had both higher median values and quartile ranges for liver function test items, including glutamic oxaloacetic transaminase (GOT) and glutamic pyruvic transaminase (GPT), than HBsAg-negative people. Proportions of the people over the cut-off values in HBsAg-positive people were 11.2% for GOT and 21.0% for GPT, which were significantly higher than those (4.0% and 11.2%) for HBsAg-negative people (p<0.001). Other clinical test results were not significantly different depending on HBsAg positive state (Table 2).

### Health behavior, mental health, and quality of life by hepatitis B surface antigen seropositivity

Indices of health behavior, mental health, and quality of life in 19-year or older adults depending on HBsAg positive state were analyzed. HBsAg-positive people with current smoking (21.2%) were higher than-negative people (19.1%), but with no significant difference. The proportions of people with high-risk drinking were 13.8% for HBsAg-positive people and 14.9% for HBsAg-negative people, showing similar levels. Moderate or vigorous physical activity rates were also similar, with 19.9% and 19.5% for HBsAg-positive and HBsAg-negative people. For mental health indices, rates of perceived stress and depressive symptoms were 23.1% and 13.4% vs. 27.4% and 12.1% for HBsAg-positive and HBsAg-negative people, with no significant differences. For the quality of life indices, when the EQ-VAS value of the healthiest state was set to 100, the values were 73.0 for HBsAg-positive and 74.2 for HBsAg-negative people, with no significant difference. With 1 as the perfectly healthy state, EQ-5D values for both HBsAg-positive and HBsAg-negative people were 0.95 without a significant difference (Table 3).

### Comorbidity and healthcare utilization by hepatitis B surface antigen seropositivity

For comorbidities, liver cirrhosis prevalence was significantly higher in HBsAg-positive people (p<0.001), whereas other diseases showed no significant differences. For healthcare utilization, there were no significant differences in experiences of outpatient, hospitalization, and cancer screening within the last 2 years, but the liver cancer screening rate of HBsAg-positive people (7.5%) was 2.3 times higher than that of negative people (p<0.001) (Table 4).

### Prevalence of liver cirrhosis by hepatitis B surface antigen seropositivity

When the combined prevalence of liver cirrhosis identified through questionnaires from 2005 to 2013 was compared, the prevalence of liver cirrhosis in HBsAg-positive people was 0.11%, which was 10 times higher than that (0.01%) of HBsAg-negative people. In particular, this difference was higher in females than in males (Table 5).

## DISCUSSION

The HBsAg seropositive rates in the Korea were decreased by 1.7% from 4.6% in 1998 to 2.9% in 2013, a 15-year period. Jeong et al. [5] studied changing trends of the HBsAg seropositivity from 1998 to 2005 using the KNHANES data, and they predicted that the HBV infection rate in the Korea would decrease remarkably in future because of the program that was introduced in 2002 to prevent vertical infection of HBV [5]; however, the seropositivity of hepatitis B in the Korea decreased to 2.9% in 2008 and has remained at a similar level for five years, ending at 2.9% in 2013.

When order three polynomial trend lines in 1998, 2005, and 2013 were added in order to compare seropositivity by age, the R2 of the order two polynomial trend lines were found to be 0.766 in 1998 and 0.775 in 2013; therefore, the order three polynomial trend lines with high R2 values (0.784 for 1998 and 0.812 for 2013) were determined to be more appropriate. According to the trend lines, age groups with the highest seropositivity were 35 years to 39 years (about 6%) in 1998 and 50 years to 54 years (about 4.7%) in 2013 after 15 years had passed, reflecting the interval between the two survey times. All age cohorts declined in infection rate by about 1% to 2% over the 15 years. Therefore, the peak of HBsAg seropositivity would be expected to gradually shift into their late 50s and 60s during the next 10 years. Considering the population structure by age, however, it is expected that 40s to 50s will remain as the main age group of patients with hepatitis B. As time passes by, seropositive rates in corresponding age cohorts are expected to change due to the effect of three factors as follows: First, increasing the number of newly infected people among susceptibles. Second, HBsAg-positive people are naturally cured or treated by drugs, resulting in becoming negative. Third, HBsAg-positive people die of liver failure such as liver cirrhosis or liver cancer. In order to reduce seropositivity in the corresponding age cohort with time, the number of reductions due to the second and third factors should become higher than that of increases due to the first factor.

In the Korea, a mass vaccination campaign against HBV has been performed beginning in the 1980s, and anyone can have vaccinations in clinics, so that the number of new infections has not been increasing significantly. Since the natural seroconversion rate that HBsAg-positive people become negative per year is known to be 0.4% [14], if it is assumed that the number of HBsAg-positive people has decreased at this rate for 15 years, it can be calculated that the 6% seropositivity in the 35 years to 39 years of age cohort in 1998 would decrease by 0.36% during the 15 years, resulting in 5.64% (Yi=seropositivity of i year, when i=1 … 15, y_i+1_=y_i_−0.004^*^y_i_).

Despite recent introduction of effective drugs for treatment of hepatitis B, their treatment rates and treatment effects have not been clearly reported in the Korea. According to the report on the third factor demonstrating that survival rates of chronic hepatitis B were 97% at the 5th year, 89% at the 10th year, and 74% at the 15th year [9], about 6% of the seropositive rate of the age cohort of 35 years to 39 years in 1998 would be 4.44% (6%*0.74=4.44%) after 15 years. Hence, considering the two reduction factors, the 6% seropositive rate would be reduced by 1.92%, resulting in 4.08%, so that it seems that about 4.5% of the current seropositive rate of the age cohort of 50 years to 54 years is attributable to a lower mortality rate of hepatitis B than in those of previous reports. However, the effect of each factor remains to be further studied.

In clinical examination results, HBsAg-positive people had significantly higher values on the liver function tests, GOT and GPT, while they showed no significant differences in other test results. HBsAg-positive people showed higher values in liver cirrhosis prevalence and liver cancer diagnosis rate. Especially, the prevalence of liver cirrhosis of HBsAg-positive people was 10 times higher than HBsAg–negative people. These results, similar to previous studies, suggest that HBsAg-positive cases are prone to morbid and chronic liver diseases.

Epidemiological studies reported 40 g to 80 g or higher ethanol per day as the alcohol amount required to cause liver damage [15-18], and alcohol drinking on HBV carriers increased the risks of liver cirrhosis and hepatocellular carcinoma [19,20]. In addition, since smoking independently increases the risks of liver cirrhosis and hepatocellular carcinoma in HBV carriers [19-21], they are recommended to avoid drinking alcohol and cease smoking [2]. Despite these risks, the results of the present study showed no significant differences in health behavior indices including smoking, drinking, and physical activities between HBsAg-positive and HBsAg-negative people. Despite a significantly higher liver cancer screening rate in HBsAg-positive people, the liver cancer screening rate of HBsAg-positive people was only 7.5%, showing poor health behavior. In addition, previous studies reported that chronic hepatitis patients that had fears of sudden aggravation of diseases and psychosocial stresses, such as withdrawal from interpersonal relationships, sense of alienation, depression, and anxiety due to incorrect social prejudices that hepatitis is highly contagious [7-9]. However, on the contrary, the current study showed no significant differences in mental health and quality of life indices. Hepatitis B patients can maintain their health conditions through regular liver function tests, timely treatments, and healthy lifestyles based on current and accurate knowledge [6]. Therefore, HBsAg-positive people need to adopt a healthier lifestyle in order to prevent progression of hepatitis B to liver cirrhosis and liver cancer.

Although the present study used nationally representative data, it was difficult to compare the prevalence of complications because of a relatively small number of HBsAg-positive people. In addition, it has the limitations of being a cross-sectional study in that identifying the order of incidents of HBsAg positivity state with risk factors was difficult. Last, although proper levels of analyzed values were obtained through internal and external controls each year, reagents and analyzers for testing HBsAg varied, so these might cause errors, although we expect them to be slight. Thus, these factors need to be considered during interpretation of the data.

In conclusion, HBsAg seropositive rates decreased from 4.6% in 1998 to 2.9% in 2008, and then persisted without significant changes, ending at 2.9% in 2013.Thus, for reduction of hepatitis B seropositive rate in the Korea, treating existing hepatitis B patients more actively appears to be necessary, along with a continuous immunization programmes to prevent new HBV infection. According to the research results in 2013, hepatitis B seroprevalence was at the highest in the early 50s, which is attributable to the shift of the peak in the mid 30s in 1998 toward an older group over the intervening 15 years. Since health behaviors did not differ between hepatitis B positive and negative people, it necessary to convince HBsAg-positive people to engage in healthier lifestyles in order to prevent the progression of hepatitis B to liver cirrhosis and liver cancer.

## Figures and Tables

**Table 1.** Laboratory method for testing hepatitis B surface antigen (HBsAg) by year

	1998	2001	2005	2007	2008-2013
Analyzer	CODA (BIORAL, USA)	Elecsys 2010 (Roche, Switzerland)	E-170 (Roche, Germany)	E-170 (Roche, Switzerland)	Modular E-170 (Roche, Germany)
Reagent	GenediaHBsAg ELISA 3.0 (GreencrossMedical Science, Korea)	Roche HBsAg (Roche, Switzerland)	Roche HBsAg (Roche, Germany)	Roche HBsAg (Roche, Switzerland)	Roche HBsAg II (Roche, Germany)

**Table 2.** Clinical characteristics by HBsAg positive status among adults≥19 years based on the KNHANES V (2010-2012), Republic of Korea

Variables	Cut-off values	Total			HBsAg-positive					HBsAg-negative				p-value
		n	%^1^	n	%^1^	Range			n	%^1^	Range			
						1Q	Median	3Q			1Q	Median	3Q	
GOT (IU/L)	>40	17,442	4.3	634	11.2	19.0	23.0	29.0	16,808	4.0	16.0	20.0	24.0	<0.001
GPT (IU/L)	>35	17,442	11.6	634	21.0	16.0	22.0	32.0	16,808	11.2	12.0	17.0	25.0	<0.001
BUN (mg/dL)	>30	17,442	0.3	634	0.5	11.0	13.0	16.0	16,808	0.2	11.0	13.0	16.0	0.20
Creatinine (mg/dL)	≥1.5	17,442	0.4	634	0.7	0.7	0.9	1.0	16,808	0.4	0.7	0.8	1.0	0.47
TC (mg/dL)	≥240	17,061	8.1	617	5.1	160.0	182.0	206.0	16,444	8.2	158.0	182.0	207.0	0.01
TG (mg/dL)	≥200	14,268	15.0	509	8.6	68.0	95.0	142.0	13,759	15.3	67.0	100.0	153.0	0.002
HDL-C (mg/dL)	<40	17,061	21.9	617	23.3	41.1	46.9	54.8	16,444	21.9	41.1	47.8	55.7	0.50
LDL-C (mg/dL)	≥100	16,736	64.6	615	66.9	92.9	110.2	132.8	16,121	64.5	91.1	111.2	132.9	0.35
Glucose (mg/dL)	≥126	17,034	5.6	615	5.9	87.0	92.0	100.0	16,417	5.6	86.0	91.0	98.0	0.82
HbA1c(%)	<4.7 or >6.9	12,052	7.0	437	5.2	5.2	5.5	5.7	11,613	7.1	5.3	5.6	5.9	0.14
Hgb (g/dL)														
Male	<13 or >16.5	17,380	14.8	632	14.7	13.3	14.5	15.6	16,746	14.8	13.0	14.2	15.4	0.95
Female	<12 or >15.5													
SBP (mmHg)	≥140	18,481	9.8	629	11.3	106.0	115.5	125.5	16,777	9.4	106.0	115.0	127.0	0.18
DBP (mmHg)	≥ 90	18,481	9.8	629	11.8	69.0	74.5	82.0	16,777	9.9	68.5	75.0	82.0	0.26
BMI (kg/m^2^)	≥25	18,371	32.0	629	35.3	21.8	23.7	25.8	16,702	32.0	21.2	23.4	25.8	0.15

All p-values were calculated by a chi-square test for the comparison of two proportions. HBsAg, hepatitis B surface antigen; KNHANES, Korea National Health and Nutrition Examination Survey; GOT, glutamic oxaloacetic transaminase; GPT, glutamic pyruvic transaminase; BUN, blood urea nitrogen; TC, total cholesterol; TG, triglyceride; HDL-C, high density lipoprotein-cholesterol; LDL-C, low density lipoprotein-cholesterol; HbA1c, glycated hemoglobin; Hgb, hemoglobin; M, male; F, female; SBP, systolic blood pressure; DBP, diastolic blood pressure; BMI, body mass index.

1 Proportion of the people over the cut-off values.

**Table 3.** Health behavior, mental health and quality of life by HBsAg seropositivity among adults ≥19 years based on the KNHANES V (2010-2012), Republic of Korea

Variables		Total	HBsAg-positive	HBsAg-negative	p-value
Smoking	Lifetime experience	16,969 (38.4)	618 (38.0)	16,351 (38.4)	0.89
	Current	16,967 (19.2)	618 (21.1)	16,349 (19.1)	0.31
Alcohol	Lifetime experience	16,972 (93.8)	618 (92.4)	16,354 (93.8)	0.18
	High-risk drinking	12,225 (14.9)	439 (13.8)	11,786 (14.9)	0.61
Exercise	Moderate to vigorous	16,946 (19.5)	616 (19.9)	16,330 (19.5)	0.86
	Moderate to vigorous (including walking)	16,923 (48.0)	614 (49.2)	16,309 (48.0)	0.61
	Vigorous	16,954 (14.2)	617 (14.1)	16,337 (14.2)	0.95
Depression	Perceived stress^1^	16,966 (27.3)	618 (25.1)	16,348 (27.4)	0.30
	Symptoms experience^2^	16,969 (12.2)	618 (13.4)	16,351 (12.1)	0.46
Quality of life	EQ-Vas	16,897 (74.2)^3^	617 (73.0)3	16,280 (74.2)^3^	0.18
	EQ-5D	16,957 (0.9)^4^	617 (0.9)4	16,340 (0.9)^4^	0.11

Values are presented as number (%). Age and sex adjusted rate. HBsAg, hepatitis B surface antigen; KNHANES, Korea National Health and Nutrition Examination Survey; HBsAg, hepatitis B surface antigen.

1 Percentage of people who usually felt ‘very much’ or ‘very’ stressed.

2 Percentage of adults who felt sad or hopeless almost everyday for two or more weeks continuously such that they stopped doing some usual activities during the past year.

3 Score ranged from 0 to 100.

4 Score ranged from 0 to 1.

**Table 4.** Comorbidity and health care utilization by HBsAg seropositivity among adults ≥ 19 years based on the KNHANES V (2010-2012), Republic of Korea

Variables	Total			HBsAg-positive			HBsAg-negative			p-value
	N	n	%	N	n	%	N	n	%	
Comorbidity										
Tuberculosis^1^	16,973	811	3.7	617	29	3.9	16,356	782	3.7	0.77
Diabetes^2^	16,596	1,718	5.2	599	43	4.0	15,997	1,675	5.3	0.16
Hypertension^3^	16,922	5,148	19.3	611	173	18.5	16,311	4,975	19.4	0.65
Hepatitis C^1^	16,971	37	0.10	616	1	0.05	16,355	36	0.11	0.44
Liver cirrhosis^1^	16,970	53	0.01	616	19	0.10	16,354	34	0.01	< 0.001
Liver cancer^1^	16,970	15	0.0001	616	5	0.001	16,354	10	0.0001	0.002
Health care utilization										
Outpatient (2 wk)	16,955	5,801	29.5	617	197	32.6	16,338	5,604	29.3	0.24
Hospitalization (1 yr)	16,955	1,834	10.8	617	67	11.1	16,338	1,767	10.8	0.84
Untreated^4^ (1 yr)	16,968	3,085	18.2	617	111	18.5	16,351	2,974	18.1	0.88
Cancer screening (2 yr)	16,964	9,265	44.9	617	360	47.8	16,347	8,905	44.8	0.28
Liver cancer screening (2 yr)	16,953	990	3.5	617	82	7.5	16,336	908	3.3	< 0.001

Age and sex adjusted rate. HBsAg, hepatitis B surface antigen; KNHANES, Korea National Health and Nutrition Examination Survey.

1 Diagnosed by medical doctor in questionnaire.

2 Proportion of people with fasting plasma glucose ≥126 mg/dL, diagnosed with diabetes by a doctor, taking oral hypoglycemic agents, or taking insulin;

3 Proportion of people with systolic blood pressure ≥140 mmHg or diastolic blood pressure ≥90 mmHg or taking anti-hypertensive agents.

4 Percentage of adults who could not visit a clinic or hospital (excluding dental clinics) when they needed during the past year.

**Table 5.** Prevalence of liver cirrhosis^1^ by HBsAg seropositivity, sex, and survey year among adults ≥ 19 years based on the KNHANES, Republic of Korea

	Survey year	HBsAg-positive			HBsAg-negative			p-value
		N	n	%	N	n	%	
Male	2005	113	1	0.01	2,138	12	0.02	0.54
	2007-2009	306	7	0.01	6,828	12	0.00	<0.001
	2010-2012	295	7	0.12	6,965	25	0.02	0.002
	2013	79	3	0.04	2,049	5	0.00	<0.001
	Subtotal	793	18	0.09	17,980	54	0.01	<0.001
Female	2005	103	0	0.00	2,982	10	0.01	<0.001
	2007-2009	311	3	0.01	9,246	5	0.00	0.001
	2010-2012	321	12	0.04	9,389	9	0.00	<0.001
	2013	86	2	0.00	2,693	4	0.00	0.007
	Subtotal	821	17	0.13	24,310	28	0.01	<0.001
Total	2005	216	1	0.01	5,120	22	0.02	0.20
	2007-2009	617	10	0.07	16,074	17	0.00	<0.001
	2010-2012	616	19	0.18	16,354	34	0.01	<0.001
	2013	165	5	0.03	4,742	9	0.00	<0.001
	Total	1,614	35	0.11	42,290	82	0.01	<0.001

Age and sex adjusted rate. HBsAg, hepatitis B surface antigen; KNHANES, Korea National Health and Nutrition Examination Survey.

1 Liver cirrhosis by questionnaire.
