# Supplementary material for: Changes in seroprevalence of hepatitis B surface antigen and epidemiologic characteristics in the Republic of Korea, 1998-2013
Source: Epidemiol Health. 2015 Dec 23;37:e2015055. doi: 10.4178/epih/e2015055 (PMC4835708; doi:10.4178/epih/e2015055)
Supplement: Supplementary file 1 [file epih-37-e2015055-supplementary.pdf]

B형 간염 표면항원 양성률 변화와 역학적 특징

(Changing Hepatitis B surface antigen seroprevalence and epidemiologic characteristics in Republic of Korea, 1998 - 2013)

Abstract

Purpose

국가수준의 B형 간염 표면항원(HBsAg) 양성률 결과가 보고된 1998년부터 2013년까지의 B형 간염 유병률 변화 추이를 살펴보고, B형 간염 양성자와 음성자의 임상적 특성, 건강행태, 삶의 질, 동반질환 및 의료이용에 차이가 있는지를 검토하고자 하였다.

Methods

국민건강영양조사 제1기부터 제6기까지 혈액검사 자료(1998-2013)를 기반으로 HBsAg 양성률을 연도별, 성별, 연령별로 비교하였다. 각 연도별 비교는 2005년 추계인구를 기준으로 연령 표준화하였다. 건강행태, 삶의 질, 동반질환 및 의료이용은 면접 및 자기기입 형식의 설문조사를 기반으로 하였다.

Results

지난 15년간 한국의 HBsAg 양성률은 1998년 4.6%에서 2008년에 2.9%로 감소한 이후 5년간 증감없이 2013년에 2.9%로 나타났다. 성별 양성률은 남자가 여자보다 높으나 그 차이는 점차 줄어드는 추세였다. 연령별 양성률은 1998년 35-39세에서 정점을 보였으나 2013년에는 50-54세가 정점을 나타냈다. HBsAg 양성자에서 간기능 검사인 GOT, GPT 수치가 높게 나타났으며, 간경화 유병률과 간암 검사율이 유의하게 높게 나타났다. 건강행태, 삶의 질 지표에서는 양성자와 음성자간에 유의한 차이를 보이지 않았다.

Conclusion

HBsAg 양성률은 1998년 이후 감소 양상을 보이다가 최근 5년간은 큰 변화를 보이지 않고 있다. 향후 우리나라 HBsAg 양성률 감소를 위해서는 새로운 B형 간염 환자의 증가를 막기 위한 예방접종사업의 지속과 함께 기존 B형 간염 환자의 치료에 보다 적극적이어야 될 것으로 보인다. 또한 B형 간염 환자의 건강행태는 음성자와 차이가 없는 것으로 나타나, 향후 B형 간염으로 인한 간경화, 간암으로의 진행을 막기

위해서는 HBsAg 양성자가 보다 건강한 생활습관을 가지도록 유도하는 것이 필요할 것이다.

Introduction

B 형 간염 바이러스(Hepatitis B virus, HBV) 감염은 간경변이나 간세포암과 같은 만성 간질환(Liver disease)의 주요 위험요인이며, 전세계적으로 발생 빈도가 높아 인류의 중요한 공중보건학적인 문제이다. 세계보건기구(WHO) 보고에 의하면 세계인구 중 약 20 억 명이 HBV 에 감염된 적이 있으며, 2015 년 기준 연간 2 억 5 천만명이 HBV 감염과 관련된 질환으로 사망한 것으로 추정된다[1]. 이러한 B 형 간염 바이러스성 간질환을 진단하는 데 B 형 간염 표면항원(Hepatitis B surface Antigen, HBsAg)을 널리 이용하고 있으며, HBsAg 양성인 경우 급성 HBV 감염으로, 6 개월 이상 검출되면 만성 B 형 간염으로 진단 할 수 있다[2]. WHO 에서는 HBsAg 양성률에 따라 1% 미만, 2-8%, 9% 이상의 지역으로 분류하고 있는데, 1980 년대 우리나라는 B 형 간염 표면항원 양성률이 8% 이상인 B 형 간염 만연 지역으로 분류되었다[3]. 그러나 B 형 간염 예방을 위한 효과적인 백신이 개발되어 우리나라에는 1983 년부터 B 형 간염 예방접종을 도입하였으며, 1995 년부터는 국가필수예방접종사업(영유아 필수 예방접종)을 실시하였다. 또한 2002 년 7 월부터는 산모에서 아기로 전염되는 수직감염을 예방하기 위하여 B 형 간염 산모의 신생아에게 B 형 간염 백신접종과 면역글로불린(Hepatitis B Immune Globulin, HBIG) 접종에 대한 예방사업 비용을 지원하고 있다[4]. 이러한 다양한 사업의 결과로 우리나라의 HBsAg 양성률은 지속적으로 감소하는 것으로 알려져 있으며, 정 등의 역시 1998 년부터 2005 년까지 우리나라 HBsAg 양성률 감소 양상에 대해 보고한 바 있다 [5]. 그러나 2005 년 이후 우리나라 B 형 간염 표면항원 양성률에 대한 보고가 없어, 최근 15 년간 B 형 간염 표면항원 양성률 추이를 살펴볼 필요가 있겠다.

B 형 간염은 일상생활관리와 함께 정기적인 간기능 검사, 시의 적절한 치료, 그리고 감염자 가족을 위한 검사와 예방접종을 통해 환자의 건강을 유지하고 전염의 고리를 끊을 수 있다. 그러므로 B 형 간염 환자는 정확한 지식에 근거하여 건강한 일상생활습관을 유지해야 함에도 불구하고, 최근의 연구에 따르면 음주, 신체활동과 같은 적절한 건강행태, 정기검진 등에 대한 불이행 정도가 높았다[6]. 또한 대부분의 만성 간염 환자들은 질병이 언제 악화될지 모른다는 두려움을 느끼며, 간염은 쉽게 전염된다는 잘못된 사회적 편견에 의해 대인관계 위축, 소외감, 우울, 불안과 같은

심리사회적 스트레스를 받고 있었다[7-9]. 따라서 이와 같은 요인들을 미리 파악하여 적절한 건강관리 전략을 마련하는 것이 매우 중요하다.

이에 이 연구는 1998 년부터 2013 년까지 국민건강영양조사(Korea National Health and Nutrition Examination Survey, KNHANES)를 통해 국가수준의 HBsAg 양성률 변화 추이를 살펴보고, HBsAg 양성 여부에 따른 임상적 특성, 건강행태, 삶의 질, 동반질환 및 의료이용을 비교하여 제시하고자 한다.

Methods

Study population

국민건강영양조사는 질병관리본부에서 수행하는 전국 규모의 건강 및 영양조사로, 국민의 건강수준, 건강행태에 대한 대표성과 신뢰성을 갖춘 통계를 산출하고 있다. 제 1 기부터 제 3 기(1998, 2001, 2005)까지 3 년 주기로 실시하였고, 이후 연중 조사체계로 개편되어 제 4 기(2007-2009)부터 현재까지 매년 실시하고 있다. 인구주택총조사자료를 추출틀로 사용하여 2 단계 층화집락표본추출을 사용하여 조사대상을 선정하여, 제 1 기부터 제 3 기까지는 약 35,000 명, 제 4 기 조사부터는 약 1 만 명을 대상으로 조사를 수행하였다. 다만 2007 년 조사는 6 개월간(7 월~12 월), 약 5 천명을 대상으로 조사를 수행하였다[10].

이 연구는 국민건강영양조사 제 1 기부터 제 6 기(1998, 2001, 2005, 2007~2009, 2010~2012, 2013) 자료를 사용하였다. HBsAg 양성률은 임상검사를 완료한 10 세 이상의 자료를 이용하여, HBsAg 검사를 시행한 최종 대상자 수는 1998 년 9,771 명, 2001 년 7,918 명, 2005 년 6,404 명, 2007 년 3,480 명, 2008 년 7,670 명, 2009 년 8,304 명, 2010 년 6,815 명, 2011 년 6,702 명, 2012 년 6,301 명, 2013 년 5,938 명이었다. 일부 임상적 특성, 건강행태, 삶의 질, 동반질환 및 의료이용 항목들은 19 세 이상에서만 조사가 되어 19 세 이상의 자료를 분석에 사용하였다.

Health examination and interview

HBsAg 결과는 대상자로부터 약 15 ml 의 혈액을 채혈하고, 30 분내 혈청분리 후 검체를 임상검사기관으로 운반하여 분석하였다. 수거된 검체는 24 시간 이내 분석을 시행하였으며, 전기적 화학발광법(Electrochemiluminescence immunoassay, ECLIA)으로 분석하였다. 분석결과의 경우, 1998 년부터 2001 년까지는 존재 여부만, 2005 년부터 2013 년은 수치로 제시되었으며[11], 결과분석 시에는 원시자료에서 제공하는 HBsAg

양성여부 변수를 사용하였다. 분석 장비 및 시약은 조사 시기에 따라 차이가 있으나(Table 1), 매년 내 · 외부 질관리를 통해 적절한 수준을 유지하였다[12].

건강설문조사 항목 중 폐결핵, C 형간염, 간경변증, 간암 질환, 의료이용 및 삶의 질 항목은 면접방법으로 조사하였으며, 흡연, 음주, 신체활동과 같은 건강행태 항목과 정신건강 항목은 자기기입식으로 조사하였다.

현재 흡연(Current smoking)은 평생 담배 5 갑(199 개피) 이상 피웠고 현재 담배를 피우는 경우로, 고위험음주(High-risk drinking)는 1 회 평균 음주량이 남자 7 잔(여자 5 잔) 이상이며 주 2 회 이상 음주하는 경우로 정의하였다. 격렬한 신체활동실천(Vigorous physical activity)은 최근 1 주일 동안 평소보다 몸이 매우 힘들거나 숨이 많이 가쁜 격렬한 신체활동을 1 회 10 분 이상, 1 일 총 20 분 이상, 주 3 일 이상 실천한 경우를 의미하며, 중등도이상 신체활동실천(Moderate to vigorous physical activity)은 격렬한 신체활동 또는 중등도 신체활동을 1 회 10 분 이상, 1 일 총 30 분 이상, 주 5 일 이상 실천한 경우로 정의하였다. 스트레스 인지율(Perceived stress)은 5 점 리커트 척도로 조사 항목 중 평소 일상생활 중에 스트레스를 '대단히 많이' 또는 '많이' 느끼는 경우를 의미하며, 우울증상경험(Feeling sad or hopeless)은 최근 1 년 동안 연속적으로 2 주 이상 일상생활에 지장이 있을 정도로 슬프거나 절망감 등을 느꼈다고 응답한 것을 의미한다. 삶의 질은 EQ-VAS 와 EQ-5D 지표를 사용하여 제시하였다. EQ-VAS 는 시각적 척도로 나타낸 주관적인 오늘의 건강상태 지표로 최저의 상태를 0, 최고의 상태를 100 으로 표현하였다. EQ-5D 는 건강관련 삶의 질의 5 가지 차원(운동능력, 자기관리, 일상활동, 통증 및 불편, 불안 및 우울)의 기술체계를 종합한 지표로 질 가중치[13]를 반영하였다.

동반질환 중 고혈압은 수축기 혈압이 140 mmHg 이상이거나 이완기 혈압이 90 mmHg 이상 또는 고혈압 약물을 복용한 경우이며, 당뇨병은 공복혈당이 126 mg/dL 이상이거나 의사진단을 받았거나 혈당강하제복용 또는 인슐린 주사를 투여받고 있는 경우를 유병으로 간주하였다. 폐결핵, C 형간염, 간경변증, 간암과 같은 동반질환은 의사로부터 진단을 받았다고 응답한 경우를 유병으로 간주하였다. 최근 2 주간 외래입원율 및 최근 1 년간 입원율, 최근 2 년간 암 검진 여부를 조사하였으며, 연간미치료율은 최근 1 년동안 본인이 병의원(치과 제외)에 가고 싶을 때 가지 못한 경우를 의미한다.

본 연구는 SAS version 9.4 (SAS Institute Inc., Cary, NC, USA)을 이용하여 복합표본분석(SURVEY PROCEDURE)을 시행하였다. 각 연도별 HBsAg 양성률은 2005 년 추계인구를 기준으로 연령 표준화하여 비교하였다. HBsAg 양성 여부에 따른 임상적 특성, 건강행태 및 동반질환 비교 결과는 원자료 수치와 가중치를 적용한 백분율을 제시하였고, 카이제곱검정 분석방법을 이용하여 통계적 유의성을 확인하였다.

Results

HBsAg seropositivity by year and sex

10 세 이상 대상자에서 HBsAg 양성률은 조사가 시작된 1998 년 4.6% (남 5.1%, 여 4.0%)였으며, 2008 년 2.9%로 감소하는 양상을 보였으나 이후 정체 양상을 보여 2013 년에는 2.9% (남 3.1%, 여 2.5%)였다. 남자의 양성률은 2010 년 2.7%로 최하값을 보였다가 2012 년에는 4.8%로 갑자기 증가하는 양상을 보였으나 나머지 연도는 전체 경향과 부합하는 양상을 보였다. 여자는 2005 년 3.1%로 크게 낮아진 후에 대체로 큰 변화 없이 2.7%에서 3.1% 사이의 양성률을 보이고 있다. 성별로는 남자가 여자보다 지속적으로 높지만 1998 년에는 그 차이가 1.1% 였으나, 2013 년에는 0.6%로 감소하였다 (Figure. 1, Supplementary Table 1).

HBsAg seropositivity by year and age

1998 년부터 2013 년까지 결과를 연령별로 비교하였다. 연령별 대상자 수가 적어 등락이 보이는 것을 줄이고 추세를 보고자 가장 적합도가 높게 나온 3 차원 추세선을 추가하였다. 각 추세선의 R^2 는 1998 년에 0.784, 2013 년에 0.8124 로 나타났다. 전체적으로 연령이 증가하면서 양성률이 증가하다 다시 감소하는 양상을 보이나 정점은 1998 년에는 30 대 후반(35-39 세)에, 2013 년에는 50 대 초반(50-54 세)에서 나타난다. 이는 두 검사의 15 년차를 반영한다고 할 수 있다. 모든 연령 코호트에서 15 년이 지나면서 약 1-2%의 양성률 감소를 보였다. 2013 년에는 50 대 후반 이후의 높은 양성률에 비해 10 대는 1%미만, 20 대는 1% 초반대의 낮은 양성률을 나타내었다 (Figure. 2, Supplementary Table 2).

Clinical test results by HBsAg

간기능 검사 항목인 glutamic oxaloacetic transaminase(GOT)와 glutamic pyruvic transaminase(GPT)의 중위수와 사분위범위가 모두 HBsAg 양성자에서 음성자보다 높게 나타났다. 이상치 기준을 넘는 사람의 비율도 HBsAg 양성자에서 GOT 와 GPT 각각 11.2%, 21.0%로 HBsAg 음성자의 4.0%, 11.2%보다 유의하게 높았다 ($p<0.001$).
신장기능검사 항목인 blood urea nitrogen(BUN), creatinine 은 HBsAg 양성 여부와 유의한 차이가 없었다. 혈중지질농도(lipid profile) 지표 중에서 total cholesterol(TC)과 triglycerides(TG)은 HBsAg 양성자와 음성자에서 중위수와 사분위수의 분포 차이는 보이지 않았으나, 기준치를 넘는 이상자 비율은 HBsAg 양성자에서 유의하게 낮게 나타났다. high density lipoprotein-cholesterol(HDL-C)과 low density lipoprotein-cholesterol

(LDL-C)은 HBsAg 양성 여부와 유의한 차이가 없었다. glucose 와 glycated hemoglobin (HbA1c)도 HBsAg 양성 여부와 유의한 차이가 없었다. hemoglobin(Hgb), blood pressure(BP), body mass index(BMI) 역시 HBsAg 양성여부와 유의한 차이가 없었다 (Table 2).

Health behavior, mental health, and quality of life by HBsAg

19 세 이상 성인에서 HBsAg 양성여부에 따른 건강행태, 정신건강, 삶의 질 지표를 비교하였다. 건강행태 지표 중 HBsAg 양성자 중 현재 흡연자는 21.2%, HBsAg 음성자 중에는 19.1%로 양성자에서 다소 높았으나, 유의한 차이는 없었다. HBsAg 양성자 중 고위험 음주자는 13.8%, HBsAg 음성자 중에서는 14.9%로 비슷하였다. 중등도 이상의 신체활동 실천율 역시 HBsAg 양성자는 19.9%, 음성자 19.5%로 비슷하였다. 정신건강 지표 중, 스트레스 인지율은 HBsAg 양성자는 23.1%, 음성자는 27.4%였으며, 우울증상 경험율 역시 HBsAg 양성자 13.4%, 음성자는 12.1%로 유의한 차이는 없었다. 삶의 질 지표 중 EQ-VAS 는 가장 건강한 상태인 100 을 기준으로 HBsAg 양성자는 73.0, 음성자는 74.2 로 유의한 차이는 없었다. EQ-5D 완전히 건강한 상태인 1 을 기준으로 역시 HBsAg 양성자와 음성자 모두 0.95 로 유의한 차이는 없었다(Table 3).

Comorbidity and healthcare utilization by HBsAg

동반질환의 경우 HBsAg 양성자에서 간경화 유병률이 유의하게 높았으나 ($p<0.001$), 그 외 질환은 유의한 차이를 보이지 않았다. 의료이용 수준도 외래, 입원, 최근 2 년 간암검진 경험 등에서 모두 유의한 차이를 보이지 않았으나, 간암 검사율만은 HBsAg 양성자에서 7.5%로 음성자의 약 2.3 배 유의하게 높았다 ($p<0.001$) (Table 4).

Prevalence of liver cirrhosis by HBsAg

설문을 통해서 간경변 진단여부를 조사한 결과, 2005 년부터 2013 년까지의 결과를 합하여 보았을 때 HBsAg 양성자의 간경변 유병률은 0.11%로 HBsAg 음성자의 0.01%에 비하여 10 배 높게 나타났다. 특히 이러한 차이는 남자보다 여자에서 크게 나타났다 (Table 5).

Discussion

Main results and the meanings

지난 15 년간 한국의 HBsAg 양성률은 1998 년 4.6%에서 2013 년에 2.9%로 약 1.7% 감소하였다. 정 등은 국민건강영양조사 자료를 이용하여 1998 년부터 2005 년까지

우리나라 HBsAg 양성률 변화 추이에 관한 연구 결과, 2002 년부터 도입되어 운영하고 있는 B 형간염 수직감염 예방사업의 결과로 향후 국내 B 형간염 바이러스 보유율은 현저히 낮아질 것으로 판단한 바 있으나 [5], 현재 우리나라 B 형간염 양성률은 2008 년 2.9%로 감소한 이후 2013 년 2.9%로 5 년간 유사한 양성률의 수준을 유지하고 있다.

연령별로 양성률을 비교하기 위하여 1998 년, 2005 년, 2013 년에 3 차원 추세선을 포함한 결과, 2 차원 추세선의 R^2 가 1998 년에 0.7658, 2013 년에 0.7754 로 나와, R^2 가 높은 3 차원 추세선이 적합하였다. 추세선으로 보았을 때 가장 양성률이 높은 연령그룹은 1998 년에는 35-39 세(약 6%) 로 나타났는데 15 년 후인 2013 년에는 50-54 세 (약 4.7%)로 나타나 두 검사기간의 차이가 그대로 나타났다. 모든 연령 코호트에서 15 년이 지나면서 약 1%-2%의 감소를 보였다. 따라서 향후 10 년간 HBsAg 양성률의 정점은 점차 50 대 후반과 60 대 등으로 옮겨가게 될 것으로 보인다. 하지만 연령별 인구구조를 고려하면 B 형 간염 환자들의 주연령대는 계속 40-50 대가 될 것이다. 시간이 지나면서 해당 연령 코호트에서 양성률이 변화하는 요인은 다음 3 가지의 영향으로 볼 수 있다. 첫째, 감수성자 중 에서 새로이 B 형 간염 양성으로 양전되어 양성자가 증가하는 경우이다. 둘째, HBsAg 양성자가 자연 치유되거나 약물치료를 통하여 음전되는 경우이다. 셋째, HBsAg 양성자가 간경화나 간암 등의 간부전으로 사망하는 경우이다. 그런데 해당 연령 코호트에서 시간이 지나면서 양성률이 감소하려면 첫째 요인에 의한 증가량보다 둘째, 셋째 요인에 의한 감소량이 훨씬 많아야 한다. 우리나라는 1980 년대부터 B 형 간염 집단예방접종을 실시하고 민간 병의원에서 누구나 백신 접종을 받을 수 있게 되면서 새로운 감염은 크게 늘어나고 있지 않는 상황이다. HBsAg 양성자가 자연적으로 음전되는 비율은 연간 0.4%로 알려져 있어 [14], 15 년간 이 속도로 감소하였다고 하면 1998 년도 35-39 세 연령 코호트의 약 6% 수준의 양성률은 15 년 후에 0.36%가 감소하여 5.64%로 감소되는 것으로 계산된다 (Yi= i 년도의 양성률이고, i = 1 … 15 일 때, $y_{i+1} = y_i - o.oo4 * y_i$). 최근 효과적인 B 형 간염 치료제가 도입되었으나 우리나라에서 B 형 간염 환자의 치료율과 치료 효과는 명확히 알려져 있지 않다. 셋째 요인으로 만성 B 형 간염의 생존율이 5, 10, 15 년째에 각각 97%, 89%, 74%로 보고 된 것을 참고하면 [9] 1998 년도 35-39 세 연령 코호트의 약 6% 수준의 양성률은 15 년 후에 4.44% (6% * 0.74 = 4.44%)가 된다. 따라서 두 가지 감소요인을 고려하면 약 6%에서 1.92%가 감소하여 4.08%의 양성률을 보여야 하므로 현재 50-54 연령의 약 4.5% 수준의 양성률은 기존 보고보다 B 형 간염의 사망률이 적어져서 나타난 결과라고 생각해 볼 수 있다. 하지만 각 요인이 미치는 영향에 대해서는 추가 연구가 필요하다.

임상검사 결과는 간기능 검사인 GOT, GPT 는 HBsAg 양성자에서 유의하게 높게 나타났으나 다른 검사결과에서는 유의한 차이가 나타나지 않았다.

HBsAg 양성자에서 간경화 유병률 및 간암 경험률이 높게 나타났다. 간경변 진단은 의사로부터 진단을 받았는지에 관한 설문응답에 근거하여 정확도가 낮을 수 있으나, HBsAg 양성자에서 음성자에 비하여 10 배 정도 높게 나타났다. 이는 기존 보고와 동일하게 본 연구에서도 HBsAg 양성일 경우 만성 간질환으로 이행될 가능성이 높음을 시사한다. 다만 단면조사 결과이기 때문에 명확한 인과관계를 설명할 수 없다는 한계를 가지고 있다. 기존 역학 연구들에서 간 손상을 유발하는 음주량은 하루 에탄올 40~80 g 이상으로 보고되었고[15-18], HBV 보유자에 대한 연구에서 음주가 간경변증, 간세포암종의 위험도를 높인다고 보고된 바 있다[19, 20]. 따라서 HBV 보유자에서 음주는 가급적 피할 것을 권장하고 있다[2]. 또한 흡연은 HBV 보유자의 간경변증 및 간세포암종의 위험도를 독립적으로 증가시키므로[19-21] 금연을 권장하고 있다[2]. 이러한 위험성에도 불구하고 이 연구결과에 따르면 HBsAg 양성자와 음성자의 흡연, 음주, 신체활동과 같은 건강행태 지표에서 유의한 차이를 보이지 않았다. 간암 검사율은 HBsAg 양성자에서 유의하게 높게 나타났으나, HBsAg 양성자의 간암 검사율도 7.5%에 그쳐 건강행태가 불량함을 알 수 있었다. 또한 기존 연구에 따르면 B 형 간염을 포함한 만성 간염환자는 질병이 언제 악화될지 모른다는 두려움을 느끼며, 간염은 쉽게 전염된다는 잘못된 사회적 편견에 의해 대인관계 위축, 소외감, 우울, 불안과 같은 심리사회적 스트레스를 받고 있었다[7-9]. 그러나 이 연구에서는 정신건강, 삶의 질 지표에서도 유의한 차이를 보이지 않았다. B 형 간염은 정확한 지식에 근거하여 건강한 일상생활 습관을 유지하면서 정기적인 간기능 검사, 시의 적절한 치료를 통해 환자의 건강을 유지할 수 있다[6]. 따라서, 향후 B 형 간염으로 인한 간경화, 간암으로의 진행을 막기 위해서는 HBsAg 양성자가 보다 건강한 생활습관을 가지도록 유도하는 것이 필요한 것으로 사료된다.

이 연구는 국가적으로 대표성 있는 자료이지만 HBsAg 양성자 수가 상대적으로 작아 합병증 등의 유병률 비교가 어려웠다. 또한 단면연구로 HBsAg 양성 여부와 동반질환, 의료이용 및 건강행태, 삶의 질 등의 선후관계를 알 수 없는 제한점이 있다. 마지막으로 매년 내 · 외부 정도관리를 통해 적절한 수준의 분석값을 확보하였지만, 그럼에도 불구하고 HBsAg 검사 시약 및 장비에 차이가 있어 이에 따른 차이가 미세하지만 있을 수 있다. 따라서 이러한 부분은 자료 해석 시에 유의해야 한다.

결론적으로 HBsAg 양성률은 1998 년도 4.6%에서 2008 년도 2.9%로 감소하였으나 이후 큰 변화가 없이 2013 년에 2.9%로 나타났다. 따라서 향후 우리나라 B 형 간염 양성률 감소를 위해서는 새로운 B 형 간염 감염을 막기 위한 백신접종사업의 지속과 함께 기존 B 형 간염 환자의 치료에 보다 적극적이어야 될 것으로 보인다. 최근 2013 년 조사결과, 50 대초반의 B 형 간염 유병률이 가장 높았는데 이는 1998 년에 30 대 중반에 정점을 이루던 양성률 분포가 15 년이 지나면서 우측으로 이동하여 50 대 초반에 정점을 이루는 분포로 변하게 되었기 때문이다. B 형 간염 환자의 건강행태는 음성자와 차이가 없는 것으로 나타나, 향후 B 형 간염으로 인한 간경화, 간암으로의 진행을 막기 위해서는 HBsAg 양성자가 보다 건강한 생활습관을 가지도록 유도하는 것이 필요한 것으로 나타났다.

Tables and Figures

Table 1. Laboratory method for testing HBsAg by year

Year	1998	2001	2005	2007	2008~2013
Analyzer	CODA(BIORAL, USA)	Elecsys 2010 (Roche, Switzerland)	E-170 (Roche, Germany)	E-170 (Roche, Switzerland)	Modular E-170 (Roche, Germany)
Reagent	Genedia HBsAg ELISA 3.0 (Greencross medical science, Korea)	Roche HBsAg (Roche, Switzerland)	Roche HBsAg (Roche, Germany)	Roche HBsAg (Roche, Switzerland)	Roche HBsAg II (Roche, Germany)

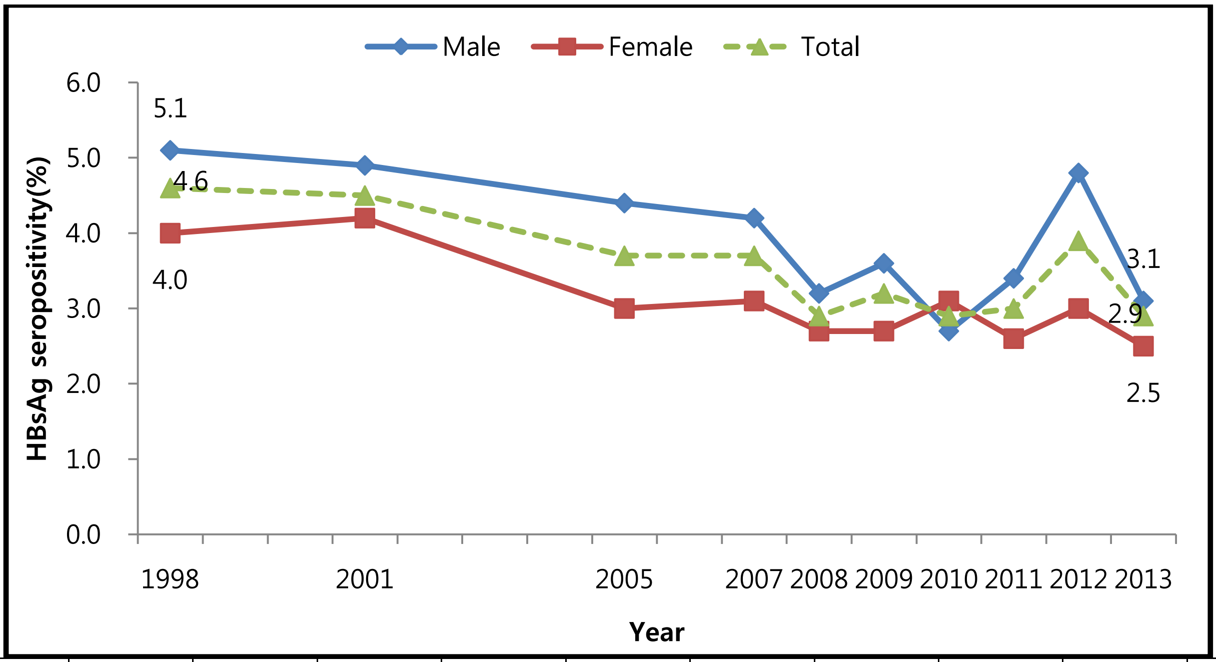

Year	1998		2001		2005		2007		2008		2009		2010		2011		2012		2013	
	N	%	N	%	N	%	N	%	N	%	N	%	N	%	N	%	N	%	N	%
Total	9,771	4.6	7,918	4.5	6,404	3.7	3,480	3.7	7,670	2.9	8,304	3.2	6,815	2.9	6,702	3.1	6,301	3.9	5,938	2.9
Male	4,514	5.1	3,568	4.9	2,826	4.4	1,511	4.2	3,357	3.2	3,724	3.6	3,064	2.7	2,956	3.4	2,754	4.8	2,670	3.1
female	5,257	4.0	4,350	4.2	3,578	3.0	1,969	3.1	4,313	2.7	4,580	2.7	3,751	3.1	3,746	2.7	3,547	3.0	3,268	2.5

Figure 1. Hepatitis B surface antigen (HBsAg) seropositivity by sex and year among ≥10 yrs based on the KNHANES I to VI (1998 – 2013), Republic of Korea.

*age standardization method is applied using the 2005 population estimates

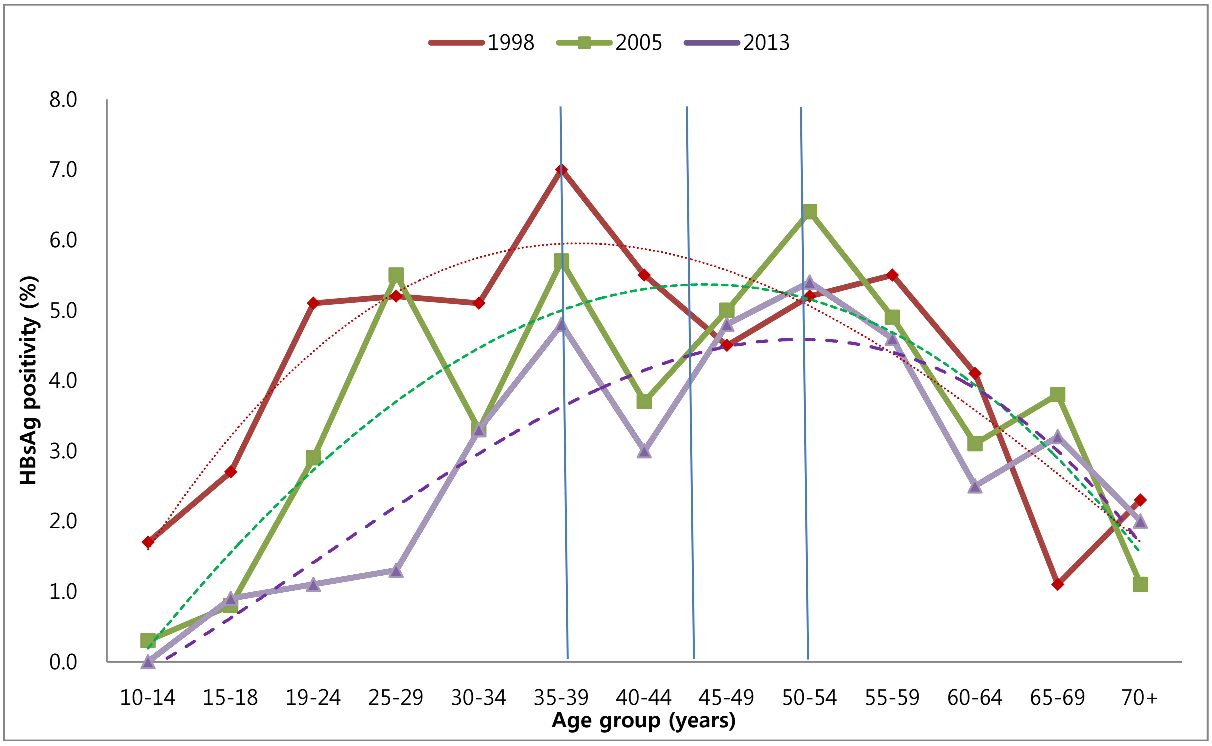

Figure 2. Hepatitis B surface antigen (HBsAg) seropositivity by agebetween 1998 and 2013, Republic of Korea.

Dotted lines are order 3 polynomial trend line: R2= 0.784 for 1998, 0.7422 for 2005 and 0.812 in 2013.

Table 2. Clinical characteristics by hepatitis B surface antigen (HBsAg) seropositivity among adult ≥19 yrs based on the KNHANES V (2010-2012), Republic of Korea

Variables	Cut off values	Total		HBsAg positive					HBsAg negative					p-value
		n	%[1]	n	%[1]	range 1Q	range median	range 3Q	n	%[1]	range 1Q	range median	range 3Q	
GOT	>40 IU/L	17,442	4.3	634	11.2	19.0	23.0	29.0	16,808	4.0	16.0	20.0	24.0	<0.001
GPT	>35 IU/L	17,442	11.6	634	21.0	16.0	22.0	32.0	16,808	11.2	12.0	17.0	25.0	<0.001
BUN	>30 mg/dL	17,442	0.3	634	0.5	11.0	13.0	16.0	16,808	0.2	11.0	13.0	16.0	0.201
Creatinine	≥1.5 mg/dL	17,442	0.4	634	0.7	0.73	0.86	0.98	16,808	0.4	0.7	0.82	0.96	0.475
TC	≥240 mg/dL	17,061	8.1	617	5.1	160.0	182.0	206.0	16,444	8.2	158.0	182.0	207.0	0.014
TG	≥200 mg/dL	14,268	15.0	509	8.6	68.0	95.0	142.0	13,759	15.3	67.0	100.0	153.0	0.002
HDL-C	<40 mg/dL	17,061	21.9	617	23.3	41.1	46.9	54.8	16,444	21.9	41.1	47.8	55.7	0.505
LDL-C	≥100 mg/dL	16,736	64.6	615	66.9	92.9	110.2	132.8	16,121	64.5	91.1	111.2	132.9	0.351
Glucose	≥126 mg/dL	17,034	5.6	615	5.9	87.0	92.0	100.0	16,417	5.6	86.0	91.0	98.0	0.824
HbA1c	<4.7 or >6.9 %	12,052	7.0	437	5.2	5.2	5.5	5.7	11,613	7.1	5.3	5.6	5.9	0.143
Hgb	M<13 or >16.5, F <12 or >15.5 g/dL	17,380	14.8	632	14.7	13.3	14.5	15.6	16,746	14.8	13.0	14.2	15.4	0.947
BP(systolic)	≥140 mmHg	18,481	9.8	629	11.3	106.0	115.5	125.5	16,777	9.4	106.0	115.0	127.0	0.185
BP(diastolic)	≥90 mmHg	18,481	9.8	629	11.8	69.0	74.5	82.0	16,777	9.9	68.5	75.0	82.0	0.261
BMI	≥25 Kg/m^2	18,371	32.0	629	35.3	21.8	23.7	25.8	16,702	32.0	21.2	23.4	25.8	0.149

All p values were calculated by chi-square test for the comparison of two proportions.

[1] Proportion of the people over the cut off values.

GOT: glutamic oxaloacetic transaminase, GPT: glutamic pyruvic transaminase, BUN: blood urea nitrogen, TC: total cholesterol, TG: triglyceride, HDL-C: high density lipoprotein-cholesterol, LDL-C: low density lipoprotein-cholesterol, HbA1c: glycated hemoglobin, Hgb: hemoglobin, BP: blood pressure, BMI: body mass index

Table 3. Health behavior, mental health and quality of life by HBsAg seropositivity among adult ≥19 yrs based on the KNHANES V (2010-2012), Republic of Korea

variables		Total		HBsAg positive		HBsAg negative		p-value
		n	%	n	%	n	%	
Smoking	lifetime experience	16,969	38.4	618	38.0	16,351	38.4	0.895
	current	16,967	19.2	618	21.1	16,349	19.1	0.306
Alcohol	lifetime experience	16,972	93.8	618	92.4	16,354	93.8	0.184
	high-risk drinking	12,225	14.9	439	13.8	11,786	14.9	0.614
Exercise	moderate to vigorous	16,946	19.5	616	19.9	16,330	19.5	0.857
	moderate to vigorous (including walking)	16,923	48.0	614	49.2	16,309	48.0	0.610
	vigorous	16,954	14.2	617	14.1	16,337	14.2	0.952
Depression	perceived stress[1]	16,966	27.3	618	25.1	16,348	27.4	0.300
	symptoms experience[2]	16,969	12.2	618	13.4	16,351	12.1	0.462
Quality of life	EQ-Vas	16,897	74.2[3]	617	73.0[3]	16,280	74.2[3]	0.182
	EQ-5D	16,957	0.95[4]	617	0.955[4]	16,340	0.950[4]	0.108

[1] Percentage of people who usually felt ‘very much’ or ‘very’ stressed.

[2]Percentage of adult who felt sad or hopeless almost everyday for 2 or more weeks continuously that they stopped doing some usual activities during the past year

[3]Score ranged from 0 to 100

[4]Score ranged from 0 to 1

*age and sex adjusted rate

Table 4. Comorbidity and health care utilization by HBsAg seropositivity among adult ≥19 yrs based on the KNHANES V (2010-2012), Republic of Korea

variables	Total			HBsAg positive			HBsAg negative			p-value
	N	n	%	N	n	%	N	n	%	
Comorbidity										
tuberculosis[1]	16,973	811	3.7	617	29	3.9	16,356	782	3.7	0.769
diabetes[2]	16,596	1,718	5.2	599	43	4.0	15,997	1,675	5.3	0.159
hypertension[3]	16,922	5,148	19.3	611	173	18.5	16,311	4,975	19.4	0.655
hepatitis C[1]	16,971	37	0.10	616	1	0.05	16,355	36	0.11	0.441
liver cirrhosis[1]	16,970	53	0.01	616	19	0.10	16,354	34	0.01	<0.001
liver cancer[1]	16,970	15	0.0001	616	5	0.001	16,354	10	0.0001	0.002
Health care utilization										
Outpatient (2 weeks)	16,955	5,801	29.5	617	197	32.6	16,338	5,604	29.3	0.239
Hospitalization (1 year)	16,955	1,834	10.8	617	67	11.1	16,338	1,767	10.8	0.844
untreated[4] (1 year)	16,968	3,085	18.2	617	111	18.5	16,351	2,974	18.1	0.884
cancer screening (2 year)	16,964	9,265	44.9	617	360	47.8	16,347	8,905	44.8	0.276
liver cancer screening (2 year)	16,953	990	3.5	617	82	7.5	16,336	908	3.3	<0.001

[1]Diagnosed by medical doctor in questionnaire

[2]Proportion of people with fasting plasma glucose ≥126mg/dL or diagnosed diabetes by a doctor or taking oral hypoglycemic agents or taking insulin

[3]Proportion of people with systolic blood pressure ≥ 140 mmHg or diastolic blood pressure ≥ 90 mmHg or taking anti-hypertensive agents

[4]Percentage of adult who could not visit a clinic or hospital (excluding dental hospital) when they needed during the past year

*age and sex adjusted rate

Table 5. Prevalence of liver cirrhosis by HBsAg seropositivity, sex and survey year among adults ≥19 yrs based on the KNHANES, Republic of Korea

		HBsAg positive			HBsAg negative			p-value
	Survey year	N	n	%	N	n	%	
Male	2005	113	1	0.01	2,138	12	0.02	0.543
	2007-2009	306	7	0.01	6,828	12	0.00	<.0001
	2010-2012	295	7	0.12	6,965	25	0.02	0.002
	2013	79	3	0.04	2,049	5	0.00	0.0003
	Subtotal	793	18	0.09	17,980	54	0.01	<.0001
Female	2005	103	0	0.00	2,982	10	0.01	<.0001
	2007-2009	311	3	0.01	9,246	5	0.00	0.001
	2010-2012	321	12	0.04	9,389	9	0.00	<.0001
	2013	86	2	0.00	2,693	4	0.00	0.007
	Subtotal	821	17	0.13	24,310	28	0.01	<.0001
Total	2005	216	1	0.01	5,120	22	0.02	0.203
	2007-2009	617	10	0.07	16,074	17	0.00	<.0001
	2010-2012	616	19	0.18	16,354	34	0.01	<.0001
	2013	165	5	0.03	4,742	9	0.00	<.0001
	Total	1,614	35	0.11	42,290	82	0.01	<.0001

Liver cirrhosis by questionnaire.

*age and sex adjusted rate

References

1.WHO. Hepatitis B[cited 2015 Jul] Available from: http://www.who.int/mediacentre/gactsheets/fs204/en.

2. Korean association for the study of the liver. 2011 KASL Guideline: management of chronic hepatitis B. Korean association for the study of the liver;2011.

3. de Franchis R, Hadengue A, Lau G, Lavanchy D, Lok A, NcIntyre N et al. EASL International Consensus Conference on Hepatitis B. 13-14 September, 2002 Geneva, Switzerland. Consensus statement(long version). J hepatol 2003;39 suppl 1:S3-S25.

4. Han JY, Jung TW, Go TG, Kim JH. A survey for changed control policies of hepatitis B in republic of korea. Korean J Pediatr Infect Dis 2011;18(2):124-134.

5. Jeong SH, Yim HW, Bae SH, Lee WC. Changes of Hepatitis B Surface Antigen Seroprevalence in Korea, 1998-2005. Korean Journal of Epidemiology 2008;30(1):119-127.

6. Choe MD, Chan N, Do HH, Woodall E, Lim E, Taylor VM. Hepatitis B and liver cancer beliefs among Korean immigrants in Western Washington. Cancer 2005;104 suppl 12:2955-2958.

7. Lee HO, Yang JH, Cho MO. Complexity and uncertainty of living with an invisible virus of hepatitis B in korea. J Canc Educ 2010;25:337-342.

8. Lee JE, Kim SS, Kim SA, Han KH, Kim SH, Ji EJ et al. Factors influencing health behavior of patients with chronic hepatitis B. Korean J Adult Nurs 2011;23(1):20-30.

9.Kim CY, Kim JW, Lee HS, Yoon YB, Song IS. Natural history and survival rate of chronic liver diseases in Korea: 20 years prospective analysis. Korean J Med 1994;46:168-180.

10. Ministry of Health and Welfare, Korea Centers for Disease Control and Prevention. Korea Health Statistics 2013: Korea National Health and Nutrition Examination Survey(KNHANES VI-1). Cheongju:Korea centers for Disease Control and Prevention;2014.

11. Ministry of Health and Welfare, Korea Centers for Disease Control and Prevention. Guidebook for data users 2013. Cheongju:Korea centers for Disease Control and Prevention;2014.
12. Korea Centers for Disease Control and Prevention. Quality control of the clinical laboratory for the korea national health and nutrition examination survey(KNHANES). Korea Centers for Disease Control and Prevention;2007-2013.
13. Korea Centers for Disease Control and Prevention. EQ-5D valuation study using time trade off method. Korea Centers for Disease Control and Prevention;2007.
14.Nam SW, Jung JJ, Bae SH, Choi JY, Yoon SK, Cho SH, et al. Clinical outcomes of delayed clearance of serum HBsAG in patients with chronic HBV infection. Korean J Intern Med 2007;22(2):73-76.
15. Ohnishi K, Terabaysahi H, Unuma T, Takahashi A, Okuda K. Effects of habitual alcohol intake and cigarette smoking on the development of hepatocellular carcinoma. Alcohol Clin Exp Res 1987;11:45-48.
16. Chevillotte G, Durbec JP, Gerolami A, Berthezene P, Bidart JM, Camatte R. Interaction between hepatitis b virus and alcohol consumption in liver cirrhosis. An epidemiologic study. Gastroenterology 1983;85:141-145.
17. Donato F, Tagger A, Gelatti U, Parrinello G, Boffetta P, Albertini A, et al. Alcohol and hepatocellular carcinoma:the effect of lifetime intake and gepatitis virus infections in men and women. Am J Epidemiol 2002;155:323-331.
18. Bellentani S, Saccoccio G, Costa G, Tiribelli C, Manenti F, Sodde M, et al. Drinking habits as cofactors of risk for alcohol induced liver damage. The Dionysoso Study Group. Gut 1997;41:845-850.
19. Chen CJ, Yang HI, Su J, Jen CL, You SL, Lu SN, et al. Risk of hepatocellular carcinoma across a biological gradient of serum hepatitis B virus DNA level. JAMA 2006;295:65-73.
20. Iloeje UH, Yang HI, Su J, Jen CL, You SL, Cen CJ. Predicting cirrhosis risk based on the level of ciuculating hepatitis B viral load. Gastroenterology 2006;130:678-686.

21. Loomba R, Yang HI, Su J, Brenner D, Iloeje UH, Chen CJ. Obesity and alcohol synergize to increase the risk of incident hepatocellular carcinoma in men. Clin Gastroenterol Hepatol 2010;8:891-898.
